# Aetiological Features of Elderly Patients with Newly Diagnosed Symptomatic Epilepsy in Western China

**DOI:** 10.1155/2018/4104691

**Published:** 2018-04-24

**Authors:** Yi Guo, Liang Yu, Baoming He, Suping Li, Qiong Zhu, Hongbin Sun

**Affiliations:** ^1^Department of Neurology, Sichuan Provincial People's Hospital, The Affiliated Hospital of University of Electronic Science and Technology of China, Chengdu, China; ^2^Department of Neurology, The Sixth People's Hospital of Chengdu, Chengdu, China

## Abstract

**Aims:**

Recent epidemiological studies have indicated that the incidence of epilepsy peaks after 60 years old, and epilepsy has become increasingly prevalent in elderly populations. The aim of this study is to identify the aetiologic characteristics of epilepsy in the elderly.

**Methods:**

We retrospectively recruited elderly patients with newly diagnosed epilepsy identified in three epilepsy centres in western China; elderly patients were defined as individuals aged 60 years or older. Demographic characteristics, clinical epilepsy data, and the diagnosis and aetiology of epilepsy were recorded.

**Results:**

A total of 760 patients with newly diagnosed epilepsy were enrolled in our study. Of these patients, 25% had experienced one or more episodes of status epilepticus, and 62.4% were confirmed as symptomatic. Among the symptomatic cohort, stroke and traumatic brain injury (TBI) were the two most common causes of epilepsy, followed by cerebral tumour, dementia, hippocampal sclerosis (HS), and central nervous system (CNS) infection. When analysed by residence and age, ischaemic stroke was the most common cause of epilepsy in urban patients, whereas traumatic brain injury was the leading cause of epilepsy in rural patients.

**Conclusion:**

More than three-fifths of newly diagnosed epilepsy cases in elderly patients were confirmed as symptomatic, and stroke and traumatic brain injury were the primary aetiologies in elderly epileptic patients.

## 1. Background

The elderly population, which comprises individuals over 60 years of age, is the most rapidly growing cohort worldwide [1]. Within the global elderly population, the elderly population in China has increased to 185 million, which accounts for 14% of the total population of China and 23% of the world's elderly population [2]. Many epidemiological studies have indicated that the incidence and prevalence of epilepsy were higher in elderly populations than in general populations [3, 4]. A survey conducted in 1993 indicated that the incidence of epilepsy increased after the age of 55 years, and the greatest incidence occurred in individuals aged 75 years or older, with an incidence rate of up to 139/100,000 [5]. Recent data have also suggested that the incidence of epilepsy in the elderly was 10 times higher than the incidence in the general population; this study indicated that among subjects aged 60–74 years, the incidence of epilepsy was 25.8/100,000, whereas in subjects aged 75 years or older, it reached as high as 101/100,000 [6].

The aetiologic features of epilepsy in elderly patients are quite different from the features in younger individuals [7, 8]. Compared with the general population, stroke and dementia have been considered to more commonly play a role in the aetiology of epilepsy in elderly populations [9, 10]. Other identified aetiologies have included but are not limited to traumatic brain injury (TBI), brain tumour, and central nervous system (CNS) infection. An established aetiology may help neurologists determine the prognosis and appropriate management of epilepsy in elderly patients. However, few studies have focused on the aetiology of epilepsy in this specific population. The aim of our study is to define the aetiologic spectrum of newly diagnosed epilepsy in patients aged 60 years or older. In this study, we found that 62.4% of epilepsy patients were confirmed as symptomatic (mostly due to stroke or traumatic brain injury), and the aetiologic spectrum differs when considering residence of the patients; traumatic brain injury is the leading cause in rural patients whereas stroke is the major aetiology in urban subjects.

## 2. Materials and Methods

### 2.1. Study Design

Sichuan Provincial People's Hospital, West China Hospital, and First Affiliated Hospital of Chongqing Medical University are tertiary referral hospitals located in Chengdu and Chongqing, China. These centres, which have authoritative epilepsy experts and are equipped with advanced electroencephalogram (EEG) and medical imaging equipment, have catchment areas that cover several regions, such as Sichuan, Chongqing, Yunnan, Guizhou, and Tibet province. These provinces have a total population of 200 million. Nearly one-fifth of the population comprised elderly individuals. We conducted a retrospective study at these epilepsy centres between January 2010 and January 2015. Patients who were newly diagnosed with epilepsy after the age of 60 years were recruited, elderly patients with epilepsy onset before the age of 60 years and an acute symptomatic seizure were excluded, and patients who received antiepileptic therapy before confirming epilepsy were also excluded. The diagnosis of epilepsy was confirmed for all subjects by two experts in this field, and abnormal CT, MRI, and EEG findings were identified by radiologists and EEG specialists. The aetiologic factors were confirmed by experts using clinical symptoms, physical examinations, and other auxiliary laboratory results. Data collected included gender, age, place of residence, age of seizure onset, disease duration, and aetiology of epilepsy. Ultimately, the data from each subject were stored in a database. We also conducted phone interviews with patients or family caregivers when necessary.

### 2.2. Diagnosis and Definitions

The diagnosis of epilepsy (idiopathic, cryptogenic, or symptomatic) was established according to the 2005 guidelines of the International League Against Epilepsy [11]. Idiopathic epilepsy was defined as having a genetic origin. Cryptogenic epilepsy was presumed to be a result of an unknown focal abnormality on the basis of an auxiliary examination. Symptomatic epilepsy was considered a consequence of definite metabolic or structural abnormalities. Aetiology was defined as the presence of a remote brain injury that causes seizure episodes [12]. Each patient was classified into one of the following aetiologic categories: stroke (ischaemic stroke, haemorrhagic stroke, or cerebral venous thrombosis (CVT)); traumatic brain injury (TBI); cerebral tumour (including glioma and meningioma); dementia (Alzheimer's disease, dementia with Lewy bodies, and other dementias); central nervous system infection; and hippocampal sclerosis (HS). Aetiologies outside of these categories were classified as “others,” such as unclear encephalomalacia, cavernous angioma, limbic encephalitis, and mitochondrial encephalomyopathy. Seizure types were classified into focal seizure and generalized seizure (tonic-clonic, absence, myoclonic, tonic, clonic, and atonic) following the revised terminology and concepts for the organization of seizures and epilepsies in 2010 by the International League Against Epilepsy (ILAE) [13]. EEG was also used as an important complementary tool to define seizure type in each patient; local or generalized paroxysms sharp waves, spike waves, sharp and slow waves, and spike and slow waves in EEG were identified as epileptiform discharges, while local or generalized slow waves in the EEG were termed as aspecific abnormality. We divided the patients into two subgroups according to age: old (younger than 75 years old) and very old (75 years old or older). The participants were divided into two groups based on patient residence: (1) urban: within 15 km of the municipal centre; (2) rural municipalities: all other areas. Status epilepticus (SE) was defined as more than 5 minutes of continuous seizure activity or intermittent seizures without fully recovering consciousness between seizures [14].

### 2.3. Ethical Approval

Our study was approved by the Medical Ethics Committee of Sichuan Provincial People's Hospital, Sichuan, China. Written informed consent was obtained from all participants or their legally appointed representatives.

### 2.4. Statistical Methods

Statistical analysis was performed using SPSS 16.0. Continuous variables were expressed as the mean and median (minimum, maximum). The aetiological prevalence was described as a proportion. Chi-square tests were used to compare subgroup variables. All *P* values were estimated using two-tailed tests. The results were considered statistically significant at an alpha value of 0.05.

## 3. Results

From January 2010 to January 2015, 760 patients with epilepsy were enrolled in our study. As shown in Table 1, 66.3% (504/760) were male, 63.9% were urban, and 579 (76.2%) were younger than 75 years. The median age of all patients was 67 years (60–96 years). The median age of seizure onset was 63 years (60–93 years), and the mean duration of epilepsy was 2 years (0–23 years). Twenty-five percent (190/760) of these patients had experienced one or more episodes of status epilepticus. Four hundred seventy-four (62.4%) patients were confirmed as symptomatic. The seizure type classifications are also shown in Table 1. Of the patients, 56.7% were diagnosed with focal seizures, 26.7% with generalized seizures, and 16.6% with unclassified seizures. All patients performed EEG recording, and epileptiform discharges were found in 20%.

Aetiologies were identified in the participants with symptomatic epilepsy.  Table 2 shows the overall frequencies of the aetiologic categories in the symptomatic cohort. Stroke was the most common cause of symptomatic epilepsy and accounted for 48.7% (*N* = 231) of the participants. Traumatic brain injury was the second most common cause of epilepsy (17.5%), and tumours and dementia each accounted for approximately 10% of epileptic aetiologies. CNS infection and hippocampal sclerosis were rare. The age of onset ranged from 60 to 70 years, and the age of onset in the patients with dementia was the highest in the cohort.

Table 3 shows the frequencies of the different aetiologies in the rural residents and urban residents in the symptomatic cohort. Of the 474 symptomatic epilepsy patients, 311 (65.6%) resided in an urban area, and 163 (34.4%) resided in a rural area. Ischaemic stroke was the leading cause of epilepsy in the urban patients, whereas traumatic brain injury was the most common epileptic aetiology in the rural patients. Moreover, the majority of patients with epilepsy caused by dementia lived in urban areas.

The aetiologies of epilepsy in the old and very old age subgroups within the symptomatic patients are shown in Table 4. These data suggest that ischaemic stroke was the main cause of epilepsy in both groups. Traumatic brain injury was the second most common aetiology associated with epilepsy in individuals younger than 75 years.

## 4. Discussion

In our study, we identified 760 elderly patients with newly diagnosed epilepsy. Of the 760 patients, 504 (66.3%) patients were male, and 256 (33.7%) patients were female; the ratio of males/females was 1.97 : 1, which was significantly higher than the gender ratios in previous studies [15–17]; however, this finding was in accordance with statistics that suggest that the majority of epilepsy patients in China were male and that the incidence of epilepsy was higher in an older male population [5, 15]. Another potential contributing factor is that males may be more likely to seek medical care because of potential gender discrimination in western China.

Status epilepticus (SE) is both a medical and neurological emergency that requires immediate recognition and prompt treatment. A survey conducted in 2016 with 340 older epilepsy patients in China suggested that approximately two-thirds had experienced SE. Our results showed that SE was confirmed in 25% of the patients, which was less common than that reported in the previous study. This discrepancy may be a result of the following causes: the incidence of SE was increased with advanced age [18, 19], while the age of our sample was younger than that of previous study. In addition, the disease duration in that paper is 3.4 ± 8.2 years and varied from 1.2 to 60.3 years, which is significantly longer than that in our study; patients with long duration in that paper have high risk of developing status epileptics.

In our sample, we identified a prevalence of focal seizures (56.7%) that was greater than that of generalized seizures and that of unclassified seizures (16.6%); this distribution was in accordance with the results of previous studies, which reported that the most frequent type of seizures in the elderly included partial seizures [5, 20]. Generalized tonic-clonic seizures may often be identified as epilepsy by most observers because of their typical, classic symptoms; however, these seizures are less common in elderly patients than in younger populations [21]. Therefore, an adaptive examination of Video-EEG for seizure classification is needed [22, 23].

Our findings suggested that stroke was the most common cause of epilepsy in ageing individuals (48.7%), and this finding was comparable to other reports in the medical literature describing both Chinese and non-Chinese populations [24, 25]. However, most published studies did not stratify the aetiologic prevalence by residence and age group. One study conducted in a cohort of 100 patients aged over 60 years in Iran recruited only participants from urban areas [26], and another survey focused solely on aetiologic differences between different age groups [27]. In our study, all the patients were categorized by both residence and age; our study suggested that more than half of epilepsy cases in urban or older groups were associated with stroke. Given the high incidence of stroke in urban areas or older populations, these results may be an accurate reflection of the characterization of epilepsy in western China. Cortical lesions and stroke severity were the independent predictor of poststroke epilepsy [28]. Therefore, urgent measures should be taken to reduce the rate of stroke through early preventative measures and lifestyle modifications to moderate the risk factors for epilepsy.

Traumatic brain injury was the leading aetiology associated with epilepsy in rural patients (27.6%) and the second most common cause in epileptic patients younger than 75 years. In contrast to previous studies that reported that TBI often occurs in older individuals because of falls, patients with TBI in our study were primarily traffic accident victims and peasant workers. However, Cheng et al. [29] found that rural residents had higher TBI mortality risk compared with urban in China (RR 2.57 versus 1.71). These findings may be a result of several factors; First, western China has a rugged terrain with mountain roads that are winding, long, and craggy. The predominant use of motorcycles in this area may be an underlying cause of the observed prevalence of TBI [30]. Second, western China also has had a substantial number of labourers relocating to the more economically developed eastern region of China, and the labourers who remain are predominantly elderly individuals younger than 75 years old in rural areas. Third, a lack of safety precautions may be a risk factor. The prognosis of traumatic brain injury is often worse in the elderly than in younger populations [31], and it has been estimated to account for one-fifth of the cases of epilepsy in ageing individuals [32]. Thus, education regarding the awareness of safety regulations is needed.

Dementia is another major risk factor for new-onset epilepsy in the elderly, and our study suggests that the age of seizure onset in patients with dementia was highest in the symptomatic cohort. Epidemiological studies have indicated that epilepsy and seizures occurred significantly more frequently in individuals with dementia, and the incidence rates of epilepsy are approximately 10 times greater in patients who suffer from Alzheimer's disease than in the general population [33]; moreover, a recent study indicated that severe cognitive impairment and younger age were independent risk factors for new-onset seizures in the elderly [34]. Our study suggested dementia was more common in urban patients, and other types of dementia were rare; these findings may be because patients who lived in the rural areas did not visit a neurologist in one of the study's tertiary referral hospitals. Lower levels of public awareness of dementia may have been another reason for the observed difference.

In our study, only 3.8% of epilepsy cases in the elderly were associated with CNS infection, and cerebral cysticercosis was the most common cause of CNS infection within this population. All patients diagnosed with cerebral cysticercosis were Tibetan; thus, these patients may have lived in endemic areas and eaten raw meat or managed livestock in an unhygienic manner. As a result of increased travel and immigration, cerebral cysticercosis may be also diagnosed in nonendemic areas [35, 36].

With the development of medical imaging techniques, an increasing number of brain tumours may be identified in the early stages. Seizures were more commonly associated with primary than secondary tumours, such as glioma and meningioma [37, 38]. Hippocampus sclerosis was less common in elderly epileptic patients, and this finding is in accordance with previous literature [39]. However, MR examination for hippocampal sclerosis is essential.

## 5. Limitations

The current study has several limitations. The most significant limitation of the current hospital-based study is the potential for a selection bias; because of stigma and traditional gender discrimination, many epileptic patients living in rural areas, particularly senior patients who live in these areas, may not visit a physician in a large tertiary hospital. In our study, the majority of senior elderly patients originated from urban areas. Second, as a retrospective study design was employed to collect data; the potential for recall bias of the patients could not be avoided, particularly regarding information obtained from participants in the very old subgroup. Third, many factors account for the aetiologic differences between rural-urban and old-very old. Age and residence cannot be independent factors. Thus, further studies are needed.

## 6. Conclusion

More than three-fifths of newly diagnosed epilepsy cases were confirmed as symptomatic in this elderly population. Stroke and traumatic brain injury were the most prevalent aetiologies of epilepsy in western China. The aetiologic spectrum varied by residence and age group. When managing epilepsy in elderly patients, the observed aetiologic distribution should be considered.

## Figures and Tables

**Table 1:** Clinical characteristics of the study population.

Variables	Recruited cases	Symptomatic
Sample size	760	474 (62.4%)
*Demographic data*		
Age, median (range)	67 (60–96)	64 (60–96)
Gender, male (*n*, %)	504 (66.3%)	306 (64.6%)
Residence, urban (*n*, %)	486 (63.9%)	311 (65.6%)
Age subgroup, old (*n*, %)	579 (76.2%)	334 (70.5%)
*Clinical data of epilepsy*		
Onset age in years, median (range)	63 (60–93)	63 (60–93)
Duration in years, median (range)	2 (0–23)	3 (0–23)
Status epilepticus (*n*, %)	190 (25%)	146 (30.8%)
*Seizure type*		
Focal (*n*, %)	431 (56.7%)	214 (45.1%)
Generalized (*n*, %)	203 (26.7%)	146 (30.8%)
Unclassified (*n*, %)	126 (16.6%)	114 (24.1%)
*EEG*		
Normal (*n*, %)	194 (25.5%)	74 (15.6%)
Aspecific abnormality (*n*, %)	414 (54.5%)	244 (51.5%)
Epileptiform discharge (*n*, %)	152 (20.0%)	156 (32.9%)

**Table 2:** Prevalence of epilepsy aetiologies among the symptomatic cohort.

Aetiology	No. of patients (*n*, %)	Age at onset (year)	
		Mean	Medians
Stroke	231 (48.7%)	68.8	66
Ischaemic stroke	175 (36.9%)	68.9	67
Haemorrhagic stroke	47 (9.9%)	68.1	65
CVT	9 (1.9%)	63.5	62
Traumatic brain injury	83 (17.5%)	62.8	60
Tumour	46 (9.7%)	64.9	61
Dementia	33 (7.0%)	70.5	69
CNS infection	18 (3.8%)	61.8	60
Hippocampal sclerosis	24 (5.1%)	65.3	61
Others	39 (8.2%)	61.8	60
Total	474 (100%)	66.4	63

**Table 3:** Aetiologic features by residence.

Aetiology	Rural		Urban		*P* value
	*N*	%	*N*	%	
Stroke	58	35.6%	173	55.6%	
Ischaemic Ischaemic stroke	39	23.9%	136	43.7%	
Haemorrhagic	13	8.0%	34	11.0%	<0.000
CVT	6	3.7%	3	0.9%	
TBI	45	27.6%	38	12.2%	
Tumour	16	9.8%	30	9.6%	
Dementia	1	0.6%	32	10.3%	
CNS infection	11	6.7%	7	2.3%	
Hippocampal sclerosis	8	4.9%	16	5.1%	
Others	24	14.7%	15	4.8%	
Total	163	100%	311	100%	

**Table 4:** Aetiologic features by age subgroup.

Aetiology	Old		Very old		*P* value
	*N*	%	*N*	%	
Stroke	137	41.0%	94	67.1%	
Ischaemic	97	29.0%	78	55.7%	
Haemorrhagic	32	9.6%	15	10.7%	<0.000
CVT	8	2.4%	1	0.7%	
TBI	74	22.2%	9	6.4%	
Tumour	32	9.6%	14	10.0%	
Dementia	17	5.1%	16	11.4%	
CNS infection	17	5.1%	1	0.7%	
Hippocampal sclerosis	19	5.7%	5	3.6%	
Others	38	11.4%	1	0.7%	
Total	334	100%	140	100%	

## Supplementary Materials

Supplementary Materials Supplementary Table 1: the distribution of other aetiology (including unclear encephalomalacia, cavernous angioma, limbic encephalitis, and mitochondrial encephalomyopathy). Supplementary Table 2: epilepsy patients with various aetiology suffered from status epilepticus.
