# Supplementary material for: Aetiological Features of Elderly Patients with Newly Diagnosed Symptomatic Epilepsy in Western China
Source: Biomed Res Int. 2018 Apr 24;2018:4104691. doi: 10.1155/2018/4104691 (PMC5941785; doi:10.1155/2018/4104691)
Supplement: Supplementary Materials — Supplementary Table 1: the distribution of other aetiology (including unclear encephalomalacia, cavernous angioma, limbic encephalitis, and mitochondrial encephalomyopathy). Supplementary Table 2: epilepsy patients with various aetiology suffered from status epilepticus. [file 4104691.f1.docx]

Supplementary table1. Distribution of other etiology

| Aetiology | Number of patients |
| --- | --- |
|  |  |
| Unclear encephalomalacia | 14 |
| Cavernous angioma | 15 |
| Mitochondria encephalomyopathy | 1 |
| Limbic encephalitis | 9 |
| Total | 39 |

Supplementary table2. Status epilepticus with vary etiology

| Aetiology | Number of patients |
| --- | --- |
|  |  |
| Cryptogenic | 56 |
| stroke | 64 |
| Ischemic stroke | 40 |
| Hemorrhagic stroke | 24 |
| Traumatic brain injury | 18 |
| Tumour | 17 |
| Dementia | 3 |
| CNS infection | 5 |
| Hippocampal sclerosis | 8 |
| Others | 19 |
| Total | 190 |
